# Supplementary material for: Modeling glioblastoma heterogeneity as a dynamic network of cell states
Source: Mol Syst Biol. 2021 Sep 16;17(9):e10105. doi: 10.15252/msb.202010105 (PMC8444284; doi:10.15252/msb.202010105)
Supplement: Supplementary file 5 — Source Data for Figure 3 [file MSB-17-e10105-s001.zip › Figure3A_sourcedata/GSEA_3065/nosigdb_state3.GseaPreranked.1622718426028/NB_KI_CLUSTER11.html]

Details for gene set NB\_KI\_CLUSTER11[GSEA]

|  || Dataset | state3 |
| Phenotype | NoPhenotypeAvailable |
| Upregulated in class | na\_neg |
| GeneSet | NB\_KI\_CLUSTER11 |
| Enrichment Score (ES) | -0.4699511 |
| Normalized Enrichment Score (NES) | -1.489468 |
| Nominal p-value | 0.029850746 |
| FDR q-value | 0.057045568 |
| FWER p-Value | 0.841 |
Table: GSEA Results Summary

  

Fig 1: Enrichment plot: NB\_KI\_CLUSTER11      
 Profile of the Running ES Score & Positions of GeneSet Members on the Rank Ordered List

  

| PROBE | GENE SYMBOL | GENE\_TITLE | RANK IN GENE LIST | RANK METRIC SCORE | RUNNING ES | CORE ENRICHMENT || 1 | B2M |  |  | 11 | 0.538 | 0.0620 | No |
| 2 | EMP3 |  |  | 91 | 0.354 | 0.0946 | No |
| 3 | CTSC |  |  | 226 | 0.272 | 0.1112 | No |
| 4 | AKAP13 |  |  | 452 | 0.209 | 0.1099 | No |
| 5 | FLNA |  |  | 633 | 0.181 | 0.1106 | No |
| 6 | MSN |  |  | 967 | 0.141 | 0.0889 | No |
| 7 | SLC2A3 |  |  | 1026 | 0.136 | 0.0983 | No |
| 8 | TMEM123 |  |  | 1047 | 0.134 | 0.1117 | No |
| 9 | CCND2 |  |  | 1286 | 0.116 | 0.0980 | No |
| 10 | THBS1 |  |  | 1561 | 0.100 | 0.0783 | No |
| 11 | IFI16 |  |  | 1576 | 0.099 | 0.0884 | No |
| 12 | ARID5B |  |  | 1833 | 0.085 | 0.0689 | No |
| 13 | SDC4 |  |  | 2189 | 0.069 | 0.0363 | No |
| 14 | BST2 |  |  | 2410 | 0.060 | 0.0181 | No |
| 15 | B4GALT1 |  |  | 3043 | 0.040 | -0.0498 | No |
| 16 | IER2 |  |  | 3345 | 0.032 | -0.0807 | No |
| 17 | IRF1 |  |  | 3457 | 0.029 | -0.0900 | No |
| 18 | PLAUR |  |  | 4075 | 0.013 | -0.1593 | No |
| 19 | TGFB1 |  |  | 4110 | 0.012 | -0.1618 | No |
| 20 | CASP4 |  |  | 4831 | -0.007 | -0.2437 | No |
| 21 | PPP1R15A |  |  | 4983 | -0.010 | -0.2599 | No |
| 22 | IFITM2 |  |  | 5106 | -0.014 | -0.2723 | No |
| 23 | GLIPR1 |  |  | 5113 | -0.014 | -0.2713 | No |
| 24 | NFKBIA |  |  | 5684 | -0.029 | -0.3334 | No |
| 25 | MBNL1 |  |  | 6042 | -0.041 | -0.3696 | No |
| 26 | SP100 |  |  | 6870 | -0.074 | -0.4559 | No |
| 27 | TGIF1 |  |  | 6888 | -0.075 | -0.4490 | No |
| 28 | FOSL2 |  |  | 6992 | -0.080 | -0.4514 | No |
| 29 | ANXA1 |  |  | 7032 | -0.083 | -0.4461 | No |
| 30 | CLIC1 |  |  | 7041 | -0.084 | -0.4372 | No |
| 31 | TSPO |  |  | 7327 | -0.102 | -0.4579 | Yes |
| 32 | ANXA11 |  |  | 7371 | -0.106 | -0.4504 | Yes |
| 33 | RARRES3 |  |  | 7473 | -0.114 | -0.4486 | Yes |
| 34 | MYL12A |  |  | 7482 | -0.115 | -0.4359 | Yes |
| 35 | NFE2L2 |  |  | 7651 | -0.133 | -0.4396 | Yes |
| 36 | EEF1D |  |  | 7817 | -0.154 | -0.4404 | Yes |
| 37 | PSMB9 |  |  | 7835 | -0.156 | -0.4240 | Yes |
| 38 | LITAF |  |  | 8015 | -0.188 | -0.4225 | Yes |
| 39 | GYPC |  |  | 8067 | -0.197 | -0.4052 | Yes |
| 40 | CIB1 |  |  | 8094 | -0.203 | -0.3844 | Yes |
| 41 | ISG15 |  |  | 8104 | -0.205 | -0.3614 | Yes |
| 42 | ARPC1B |  |  | 8131 | -0.212 | -0.3394 | Yes |
| 43 | PNRC1 |  |  | 8133 | -0.212 | -0.3145 | Yes |
| 44 | S100A11 |  |  | 8490 | -0.336 | -0.3159 | Yes |
| 45 | SAT1 |  |  | 8533 | -0.354 | -0.2792 | Yes |
| 46 | S100A10 |  |  | 8717 | -0.558 | -0.2346 | Yes |
| 47 | S100A6 |  |  | 8729 | -0.615 | -0.1635 | Yes |
| 48 | PFN1 |  |  | 8732 | -0.624 | -0.0903 | Yes |
| 49 | SH3BGRL3 |  |  | 8746 | -0.785 | 0.0006 | Yes |
Table: GSEA details [plain text format]

  

Fig 2: NB\_KI\_CLUSTER11: Random ES distribution      
 Gene set null distribution of ES for **NB\_KI\_CLUSTER11**
